# Case of Epilepsy

**Published:** 1856-12

**Authors:** J. J. Ellison

**Affiliations:** Primrose, Iowa


### A CASE OF EPILEPSY.

BY J. J. ELLISON.

W. M., a lively dispositioned, light complexioned lad, eight years of age, came under treatment for the above disease in Nov., 1855. Having learned the history and obtained the leading features of the case previously, and believing therefrom, the prognosis to be doubtful, Dr. Atkinson, of Dover, Iowa, was requested to see the patient with me on my first visit, when, on examination and inquiry, we found the circumstances and symptoms to be substantially as follows:

There is no manifest physical deformity, nor intellectual nor moral peculiarity, either congenital or acquired, and no hereditary predisposition to disease known. Five years before had suffered trom an attack of Meningitis, followed with inflammation and purulent discharge from both the nasal and aural cavities, which continued for a year or more. The first "fit" occurred about the time of its suppression; the second one a year afterward, since when, they had steadily increased in frequency of recurrence. The patient had resided in this vicinity for about four years. He had taken oil of turpentine, "whisky and assafœdita," and had used the cold shower bath with no perceptible benefit. The appetite is good; no organic lesion evident; slight paralysis ot the upper and lower extremities of the left side; there is more or less somniloquy each night.

The paroxysms recurred without any previous warning, except a light "hammering" in his head; their accession is not accompanied by any "cry," but as they pass off are attended with short struggling efforts to that purpose which end with the fits in sobbing. During the time the convulsion continues, his breathing is embarrassed, his face turgid, and the limbs of the left side as also the same side of the face, severely effected with clonic spasm. After the paroxysm has subsided, he is left heavy, stupid and partially exhausted for a few minutes, after which he appears perfectly well until another seizure takes place, which is as often as once an hour.

The conclusion arrived at by us, as to the cause of the disease, was, that it resulted from a morbid condition; constituting irritation of the base of the brain, induced by the attack of Menningitis and an unguarded suppression of the discharge from the sinuses referred to.

To remedy this condition and remove the epileptic habit, we agreed upon the trial of a tonic treatment, in conjunction with counter irritation, anti-spasmodics, and the use of galvanism. The agents to be used were zinc, iron, belladonna, an issue or a seton in the back of the neck, and a common galvanic battery. Before commencing with them, however, it was also agreed that the case should be referred for further consideration to Prof. McGugin, of Keokuk, Iowa, who, in connexion with Prof. Allen, of the same place, after a careful examination, concurred in the opinion already given, both as to the diagnosis and indications of treatment, proposing the following articles and formula, as being most adapted to secure the required end:

Of metallic tonics, Hydrocyanate or the Lactate of Iron, valerianate of zince, with belladona, extract of valerion, as proposed on page 472 of the *Iowa Medical Journal*, Vol. 2. A small seaton is to be introduced into the nape of the neck, to keep up a constant counter-irritation, and an errhine to re-establish the discharge from the nasal sinuses, with the use of the galvanism appled in silent stream through the head and spine.

Nov. 22d, '55. The treatment adopted consisted of a combination of the articles mentioned, and put up after the following formula:

℞. Valerianate of zinc, ʒii.

Lactate of Iron, ʒiss.

Extract of Belladona, ʒss.

Simple Syrup, q. s. to form into a mass.

Divided into 120 pills and take one every eighteen hours for first three days, after that, gradually increased to one every twelve hours, and then continued at that interval. A seaton of a half dozen silk threads was inserted into the back of the neck with large flat surgical needle, and dressed every day with cantharadin cerate, until the necessary amount of irritation is produced, which is to be kept up afterward by moving it as often as necessary for that purpose. An errhine of common tobacco snuff, is to be used four or five times a day or oftener; and the galvanism is applied in the manner proposed, the diet was recommended to be generous, and bowels regulated with castor oil. For several days, after commencing this course of medication, there was no apparent improvement; in fact, the symptoms, in some respects, were evidently for the worse, the paralysis being so much greater that the use of the extremities so effected, was almost entirely lost, but within the three weeks after it was commenced, the interval between the paroxysms had increased, and the convulsions had become less violent, and by the first of January following, had ceased to recur; and since then he has not suffered a single paroxysm up to the present time. He is now entirely free from all symptoms or effects of the disease, having become entirely relieved from the paralysis during the three weeks following the last convulsion, during which time tho treatment was gradually withdrawn.

Primrose, Iowa, July 22d, 1856.

